# Network Pharmacological Prediction and Experimental Analyses Reveal That Naringin Alleviates Osteoarthritis Progression by Targeting MMP13

**DOI:** 10.33549/physiolres.935675

**Published:** 2026-02-01

**Authors:** Min LI, Yunshan YAO, Donglan HUANG, Jiao DAI

**Affiliations:** 1 Department of Pharmacy, Chongqing Changshou Women & Children’s Hospital, Chongqing, China; 2 Department of Pharmacy, Chongqing University Affiliated Renji Hospital (The Fifth People’s Hospital of Chongqing), Chongqing, China; 3 Department of Breast and Thyroid Surgery, People’s Hospital of Changshou Chongqing, Chongqing, China; 4 Department of Pharmacy, People’s Hospital of Changshou, Chongqing, China

**Keywords:** Osteoarthritis, Naringin, Network pharmacology, MMP13, Molecular mechanism

## Abstract

Osteoarthritis (OA) is a severe chronic inflammatory disorder with limited treatment options. Naringin (nar) has been shown to protect against OA; however, its mechanisms of action on OA remain poorly understood. This study aims to investigate the molecular mechanism of nar in treating OA *via* network pharmacology and experiments. Differentially expressed genes (DEGs) were identified using GSE283079 dataset. Protein-protein interaction (PPI) network was constructed using STRING database, and protein interactions were analyzed. Network pharmacology was employed to investigate the molecular interaction network influenced by nar in OA, and molecular docking was applied to predict the binding interactions between nar and core genes. The OA mouse models were constructed using anterior cruciate ligament transection (ACLT) to explore the action of nar *in vivo*. The OA damage was examined using Hematoxylin and Eosin (HE) and Safranin-O/Fast Green staining, along with Osteoarthritis Research Society International (OARSI) scoring for quantitative histopathological evaluation. Terminal deoxynucleotidyl transferase dUTP nick end labeling (TUNEL) positive rate and inflammation factor (tumor necrosis factor (TNF)-α and interleukin (IL)-1β), and reactive oxygen species (ROS) levels were detected using corresponding assay kits. The protein expression was analyzed using western blot. Cell viability and cell apoptosis were examined using cell counting kit 8 (CCK8) assay kit and flow cytometry assays. In GSE283079 dataset, the up-regulation of DEGs was enriched in immune response activation, cartilage development, collagen metabolic process, and leukocyte proliferation. Additionally, matrix metalloproteinase 13 (MMP13), MMP1, and phospholipase A2 group IIA (PLA2G2A) may be the core genes for nar-protected OA. The binding energy of nar and MMP13 was strongest. *In vivo* OA models, nar mitigated OA progression and reduced OARSI scores. Mechanistically, nar suppressed cell apoptosis, inflammation factor productions, extracellular matrix (ECM) degradation, and ROS production *via* decreasing MMP13. Nar alleviates OA malignant progression *via* reducing MMP13.

## Introduction

Osteoarthritis (OA) is a common chronic degenerative joint disease and a type of arthritis characterized by damage to joint cartilage or the underlying subchondral bone, which can induce severe pain and permanent joint damage, affecting approximately 500 million adults worldwide [1,2]. The hallmark symptom of OA is joint pain, which serves as the main reason for medical consultations and often results in functional limitations and diminished quality of life [3]. Currently, there is no effective treatment for OA. Therefore, it is necessary to research and develop promising new therapies for OA.

Naringin (nar), a flavonoid compound isolated from citrus fruits, possesses a chemical structure consisting of a flavonoid backbone with two phenolic rings. Notably, it is characterized by prominent antioxidant and anti-inflammatory properties [4]. For instance, Huang *et al.* demonstrated that nar alleviated severe lung injury induced by Actinobacillus pleuropneumoniae *via* decreasing excessive inflammatory responses and increasing antioxidant capacity [5]. Zhao *et al.* showed that nar could protect against cartilage destruction in OA through inhibiting the nuclear factor kappa-B signaling pathway [6]. Additionally, nar could decrease tumor necrosis factor (TNF)-α, prostaglandin E2, and interleukin (IL)-6 expression, preventing the development of OA [7]. In summary, nar is related to OA progression.

Network pharmacology integrates molecular biology, conventional pharmacology, and bioinformatics to explore the mechanisms by which drugs regulate disease by creating network connections between disease, targets, and Drugs [8–10]. Molecular docking serves as a computational predictive tool to predict the relationship between target molecules and Drugs [11]. Furthermore, molecular dynamics simulations provide dynamic insights into drug-target interactions, elucidating binding mechanisms, complex stability, and interaction patterns. The combination of molecular docking and molecular dynamics simulations allows for comprehensive analysis of the binding stability between key target molecules and active compounds [12]. Therefore, the objective of this study is to clarify how nar influences the progression of OA through utilizing network pharmacology, molecular docking, and experimental validation.

## Materials and Methods

### Bioinformatics

GSE283079 dataset was obtained from GEO database (www.ncbi.nlm.nih.gov/geo/). Differentially expressed genes (DEGs) were screened according to P<0.05 and |log2FC|>1. Subsequently, volcano maps were constructed by the online platform (https://www.bioinformatics.com.cn/). Additionally, GO analysis was performed according to up-regulation DEGs using the R Package clusterProfiler.

### Network pharmacology analysis

The nar’s structure was downloaded from PubChem (https://pubchem.ncbi.nlm.nih.gov/). After that, the Swiss Target Prediction database (http://www.swisstargetprediction.ch/) was conducted to analyze the target of nar. OA-related target genes were predicted using GeneCard database (https://www.genecards.org/). Following that, the Venn diagram of overlapping targets was constructed using micro Sheng letter online website (https://www.bioinformatics.com.cn/). STRING database (https://string-db.org/cgi/input.pl) was performed to construct the protein-protein interaction (PPI) network.

### Molecular docking simulation

The chemical nomenclature, three-dimensional (3D) conformation, and molecular mass of nar were obtained from the PubChem database (https://pubchem.ncbi.nlm.nih.gov/). The 3D structures of the proteins matrix metalloproteinase (MMP) 13, MMP1, and phospholipase A2 group IIA (PLA2G2A) were retrieved from the Structural Bioinformatics Protein Data Bank database (http://www.rcsb.org/). Afterwards, molecular docking was conducted by AutoDock Vina software (http://vina.scripps.edu/). The crystal structures of the target biomarkers were prepared by eliminating amino acid modifications, water molecules, and hydrogenation, followed by adjusting the force field and optimizing energy parameters. The modified structures of these two molecules were saved as pdbqt format files and re-imported into AutoDockTools software as ligands and receptor respectively. Finally, AutoDock Vina was run, and then the results were visualized using PyMOL software.

### Animal experiments

OA mouse models (OA group) were constructed using anterior cruciate ligament transection (ACLT) [13]. Spiff Biotechnology Co., Ltd. (Beijing, China) offered the C57BL/6 mice, and the mice were randomly divided into three groups following the purposes of experiment. For the sham group, the mice’s joint capsules were opened and stitched to simulate surgery; in the nar (≥98 %) (Baoji Herbest Bio-Tech Co., Ltd., Baoji, China) treatment group, 100 mg/kg nar was orally administered once a day for 4 weeks after bilateral anterior cruciate ligaments were transected 2 weeks later [6]. In the sham groups, the mice were given oral administration of equal volume PBS. Four weeks after surgery, the mice were killed and knee tissue and serum were collected. The experimental operation in this research was performed in strict accordance with the guidelines of the Animal Care and Use Committee of Chongqing Changshou Women & Children’s Hospital.

### Hematoxylin and Eosin (HE) and Safranin-O/Fast Green staining

The knee tissue was fixed in 10 % formalin (Kanglang Biotechnology Co., Ltd., Shanghai, China) and embedded in paraffin. The sections (4 μm) were dewaxed, rehydrated and stained with HE (Beyotime, Shanghai, China) or Safranin-O/Fast Green (OriGene Technologies, Rockville, USA) according to the corresponding guidelines of manufacturer. Finally, the OA damage was analyzed using a microscope (Jiapeng Technology Co., Ltd., Shanghai, China). Osteoarthritis Research Society International (OARSI) score was performed to score the stained sections [14].

### Apoptosis ability detection

Terminal deoxynucleotidyl transferase dUTP nick end labeling (TUNEL) assay kit was acquired from Beyotime. Briefly, xylene was used to dewax sections of knee tissue. Then the slices were contacted with anhydrous ethanol (Kanglang Biotechnology Co., Ltd), 90 % ethanol (Kanglang Biotechnology Co., Ltd), 70 % ethanol, and distilled water. The slices were reacted with protease K (Thermo Fisher Scientific, Waltham, MA, USA) without DNase for 0.5 h at 37 °C. Following that, the slices were reacted with TUNEL detection solution at 37 °C in a dark environment for 60 min. The slices were incubated with DAPI solution (Thermo Fisher Scientific) for 6 min. Finally, the TUNEL positive rate was observed using a fluorescence microscope (Jiapeng Technology Co., Ltd.).

### Detection of TNF-α and IL-1β productions

ELISA assay kits (Beyotime) were used to examine the TNF-α and IL-1β levels. The serum of mice or cell supernatants of different treatments was collected, and then the experimental operation was performed according to the recommendation of manufacturer. Lastly, the microplate reader was conducted to test the releases of TNF-α and IL-1β.

### Detection of protein levels

The protein was extracted from cells with different treatments and knee tissues using RIPA solution (Beyotime). Subsequently, BCA technique was conducted to examine the protein concentration in the samples. Sodium dodecyl sulfate-polyacrylamide gel electrophoresis was used to separate the total proteins. The proteins were shifted to the polyvinylidene fluoride membrane (Vazyme) and then combined with primary antibodies for overnight at 4 °C. Next, the membranes were reacted with Goat Anti-Rabbit IgG H&L (HRP) (ab6721, 1:20000, Abcam) for 2 h at 37 °C. Ultimately, the results were observed using ECL reagent (Vazyme). Primary antibodies were listed as blow: anti-MMP13 (ab39012, 1:6000, Abcam, Cambridge, MA, USA), anti-recombinant a disintegrin and metalloproteinase with thrombospondin 5 (ADAMTS5) (ab41037, 1:250, Abcam), β-actin (ab8227, 1:5000, Abcam), anti-Reduced type II collagen alpha 1 (COL2A1) (sc-52658, 1:1000, Jinpan Biotechnology Co., Ltd., Shanghai, China), and anti-Aggrecan (ab315486, 1:1000, Abcam).

### Cell culture

C-28I2 cells were acquired from Green Flag Biotechnology Development Co., Ltd. (Shanghai, China). In addition, the cells were grown in Dulbecco’s modified Eagle medium (Beyotime) containing 10 % fetal bovine serum (Beyotime) and 1 % penicillin/streptomycin (Vazyme).

### Cell treatment and transfection

To stimulate an inflammatory response, the cells were exposed to IL-1β (10 ng/ml; Sigma-Aldrich, St. Louis, MO) for 24 h.

IL-1β (10 ng/ml)-induced cells were stimulated with nar (0.5, 2.5, 5, or 7.5 μM) for 24 h, or the cells were exposed to 5 μM nar for 12, 24, or 36 h. For function assays, cells were exposed to 5 μM nar for 24 h after cells were exposed to IL-1β.

The MMP13 overexpression vector (MMP13) and negative control (vector) were constructed by Wuhan Genecreate Biological Engineering Co., Ltd. (Wuhan, China). The C-28I2 cells were divided into five groups and named Control (C-28I2 cells were treated with DMSO), IL-1β (C-28I2 cells were stimulated with 10 ng/ml for 24 h), IL-1β+nar (after C-28I2 cells were exposed to IL-1β (10 ng/ml), the cells were treated with 5 μM nar for 24 h), IL-1β+nar+vector (after cells were transfected with vector, the cells were stimulated with IL-1β (10 ng/ml) and 5 μM nar), and IL-1β+nar+MMP13 (cells were stimulated with IL-1β (10 ng/ml) and 5 μM nar after cells were transfected with MMP13 overexpression vector). Lipomaster 3000 Transfection Reagent (Vazyme) was carried out to transfect the cells with plasmid.

### Measurement of cell viability

The cell viability was examined using the cell counting kit 8 (CCK8) assay kits (Beyotime). Shortly, the cells were incubated in 96-well microplates, followed by treatment with various regimens according to specific experimental purposes. Subsequently, the CCK8 detection solution was added into each well of 96-well microplates and reacted for 2 h in a 5 % CO_2_ incubator at 37 °C. The microplate reader was to examine optical density.

### Flow cytometry

Following the respective treatments, cells were harvested. After washing, the cells were re-suspended using a binding buffer (KeyGen Biotech, Nanjing, China). Subsequently, the cell suspension was reacted with Annexin V-FITC and PI solution for 15 min. Ultimately, cell apoptosis was examined using a flow cytometry detection system.

### Reactive oxygen species (ROS) detection

The ROS productions were analyzed by ROS assay kits (Enzyme Linked Biotechnology Co., Ltd., Shanghai, China). In brief, the cells were plated into 6-well microplate. Following that, the cells were stimulated with different treatments and named the Control, IL-1β, IL-1β+nar, IL-1β+nar+vector, and IL-1β+nar+MMP13 groups. The 2′,7′-Dichlorodihydro-fluorescein diacetate (DCFH-DA) was added into each well and reacted for 0.5 h at 37 °C. Finally, the results were assessed using a fluorescence microscope.

### Data analysis

All data of results were shown as mean ± standard deviation. Additionally, each experiment was repeated at least three times. GraphPad Prism 8 software (GraphPad Software, Boston, MA, USA) was performed for statistical analysis of all experimental data. The difference among three or more groups was analyzed by analysis of variance (ANOVA) followed by Tukey’s test. *P*<0.05 was regarded as the statistical significance.

## Results

### Screening of GSE283079-DEGs and enriched terms

**Fig. 1:**
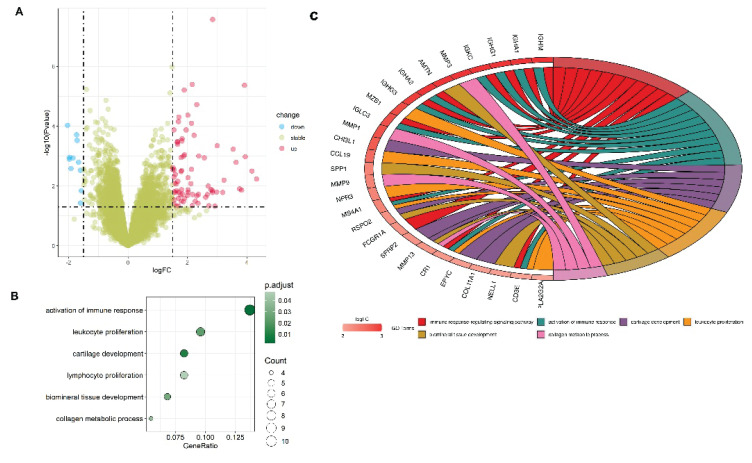
Screening of GSE283079-DEGs and enriched terms. (**A**) Volcano map of DEGs. Red dots on the right indicate genes with elevated expression, and blue dots on the left indicate genes with reduced expression. (**B**) Upregulation DEGs GO analysis. (**C**) Chord diagram illustrating the association between up-regulated DEGs and enriched GO biological process terms. Genes were listed on the left, and GO terms were shown on the right. Colored ribbons connect genes to their enriched GO terms, with colors corresponding to specific GO terms as indicated in the legend.

The volcano map of DEGs in GSE283079 dataset was shown in Fig. 1A. The GO analysis results demonstrated that up-regulation of DEGs (top six enrichments) was enriched in activation of immune response, cartilage development, collagen metabolic process, and leukocyte proliferation (Fig. 1B). As shown in Fig. 1C, the results showed that multiple key genes (MMP13, R-spondin 2 (RSPO2), and so on) were involved in collagen metabolic process, cartilage development, and leukocyte proliferation.

### Network pharmacological analysis of nar for treatment of OA

**Fig. 2:**
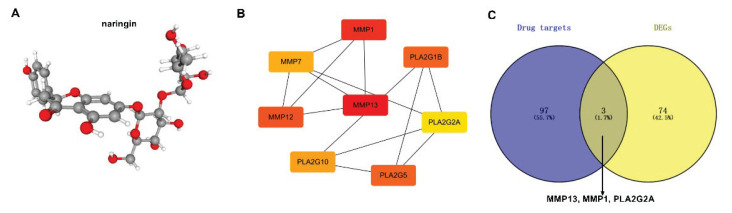
Nar may act on OA by targeting core targets MMP13, MMP1, and PLA2G2A. (**A**) The structure of nar (ball-and-stick models). (**B**) PPI network of hub targets. (**C**) Venn diagram was used to show the hub genes between OA targets and nar targets.

Next, we investigated the therapeutic targets involved in the nar treatment of OA by network pharmacology. The molecular structure of nar is shown in Fig. 2A. The PPI results showed that MMP13 and MMP1 play an important role in treatment of OA by nar (Fig. 2B). Besides, the hub genes were identified in 77 genes related to OA and 100 nar-related genes, the results demonstrated that there were 3 overlapping genes (MMP13, MMP1, and PLA2G2A) (Fig. 2C). Taken together, nar may participate in the treatment of OA.

### Results of molecular docking

**Fig. 3:**
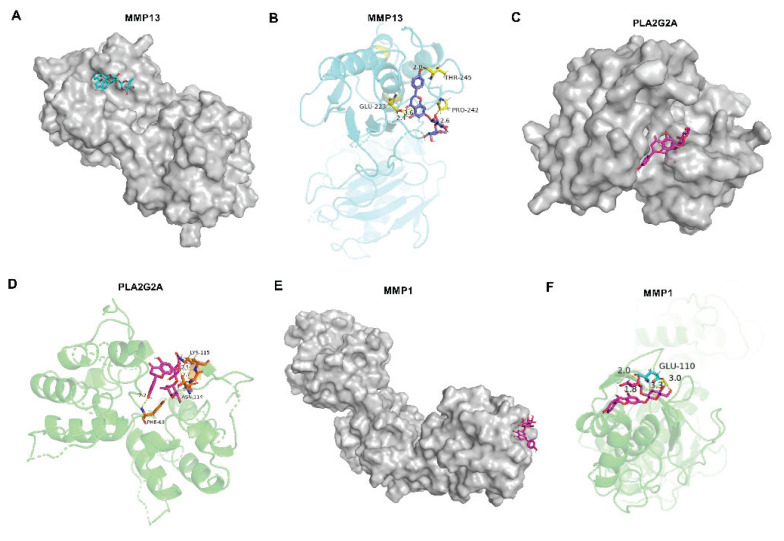
The docking model of nar with MMP13, PLA2G2A, and MMP1, respectively. Each docking result was presented in two diagrams. (**A–B**) The action model of nar with MMP13 was analyzed *via* molecular docking. (**C–D**) The interaction between nar and PLA2G2A was predicted. (**E–F**) Molecular docking was used to analyze the model of nar with MMP1.

Subsequently, we performed molecular docking of three core key genes (MMP13, MMP1, and PLA2G2A). The binding energy of nar and MMP13 was −9.9 kcal/mol (Fig. 3A, B), and the binding energy of nar and PLA2G2A was −9.3 kcal/mol (Fig. 3C, D). Additionally, the molecular docking result of nar and MMP1 was shown in Fig. 3E and F, the binding energy of nar and MMP1 was −9.2 kcal/mol. Notably, the binding energy of nar and MMP13 was strongest. Thus, researchers suggest that nar alleviates OA through targeting MMP13.

### Nar suppresses disease progression in ACLT-induced OA

**Fig. 4:**
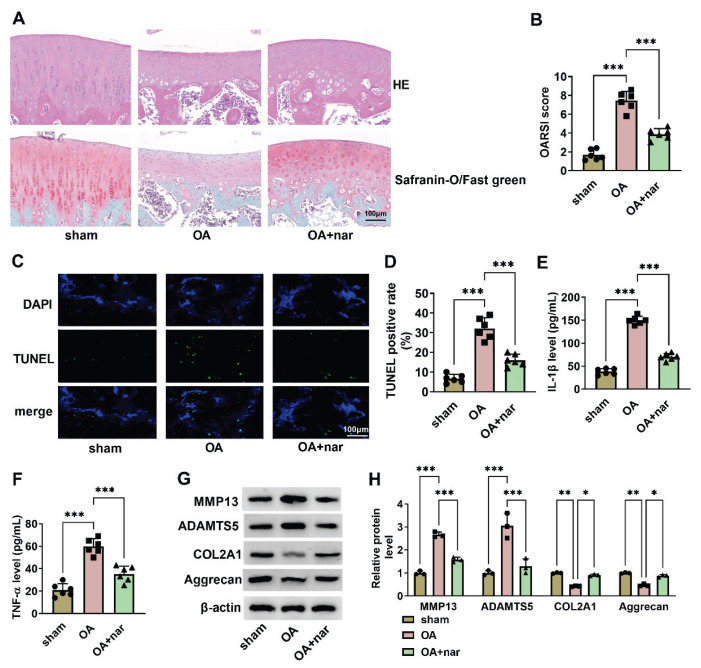
In mice models, nar alleviates OA progression. The mice were classified into three groups, namely sham group, OA group, and OA+nar group. (**A**) The OA damage was analyzed using HE and Safranin-O/Fast Green staining. (**B**) The OARSI score was analyzed. (**C–D**) TUNEL assay was used to examine the TUNEL positive rate. (**E–F**) The TNF-α and IL-1β levels were tested using corresponding ELISA kits. (**G–H**) The MMP13, ADAMTS5, COL2A1, and Aggrecan levels were detected using Western blot. * *P*<0.05, ** *P*<0.01, and *** *P*<0.001.

Authors constructed the OA models *in vivo* to explore the action of nar in OA progression. In the OA group, the cartilage was degraded and synovial membranes proliferated, which was weakened by nar (Fig. 4A). Compared with the sham group, OARSI score was significantly elevated in OA group, but nar treatments weakened this action (Fig. 4B). In OA group, TUNEL positive rate was increased when compared with the sham group, which was abolished by nar (Fig. 4C, D). In the OA group, IL-1β and TNF-α levels were promoted compared with the sham group, while nar weakened these effects (Fig. 4E, F). Notably, ECM protein-degrading enzymes (MMP13 and ADAMTS5) were increased, and ECM protein (COL2A1 and Aggrecan) levels were reduced in OA group, which was abolished by nar (Fig. 4G, H). Overall, nar can partially reverse ECM degradation, apoptosis, and inflammation in ACLT-induced OA mice.

### Nar mitigates inhibition of cell viability in IL-1β-stimulated C-28I2

**Fig. 5:**
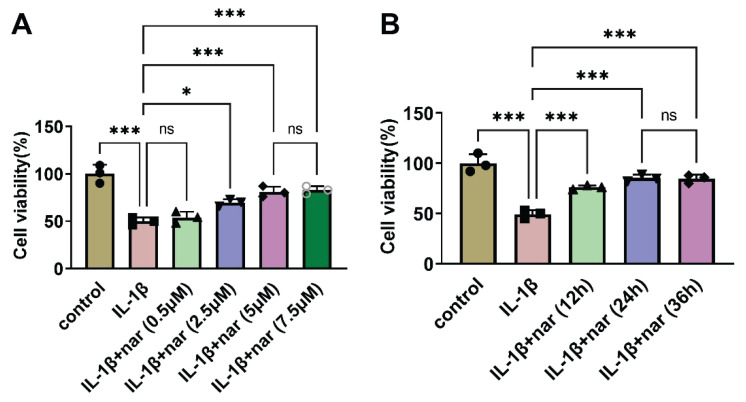
In IL-1β-stimulated C-28I2 cells, nar can promote cell viability. The C-28I2 cells were stimulated with DMSO or IL-1β (10 ng/ml) for 24 h. (**A**) IL-1β-induced C-28I2 cells were pre-incubated with IL-1β (10 ng/ml) for 24 h before exposure to various concentrations of nar (0.5, 2.5, 5, or 7.5 μM) for 24 h. The cell viability was analyzed using CCK8 assay. (**B**) IL-1β-induced C-28I2 cells were treated with nar (5 μM) for 12, 24, or 36 h. The cell viability was detected by CCK8 assay. * *P*<0.05, *** *P*<0.001, and ns indicates no significant difference.

Next, IL-1β (10 ng/ml) was employed to stimulate cells for 24 h, mimicking exposure to pro-inflammatory stimuli. As shown in Fig. 5A, IL-1β hindered cell viability when compared with the control group; compared with the IL-1β group, nar (0.5 μM) did not alter cell viability, but nar (2.5, 5, and 7.5 μM) significantly promoted cell viability; notably, nar (7.5 μM) did not change the cell viability when compared with the IL-1β+nar (5 μM) group. Therefore, this study selected the nar (5 μM) regarded as the object concentration to explore the optimal processing time. The results demonstrated that nar (5 μM) weakened IL-1β-induced inhibition on cell viability in a time-dependent manner (12 and 24 h), but when IL-1β-induced cells were treated with nar (5 μM) for 36 h, the cell viability did not alter when compared with the IL-1β+nar (24 h) (Fig. 5B). Above, researchers selected nar (5 μM) and 24 h to stimulate the cells and to explore the role of nar in biological function of OA.

### In IL-1β-induced C-28I2 cells, nar inhibits cell apoptosis, inflammation factor production, ECM degradation, and ROS production via silencing MMP13

**Fig. 6:**
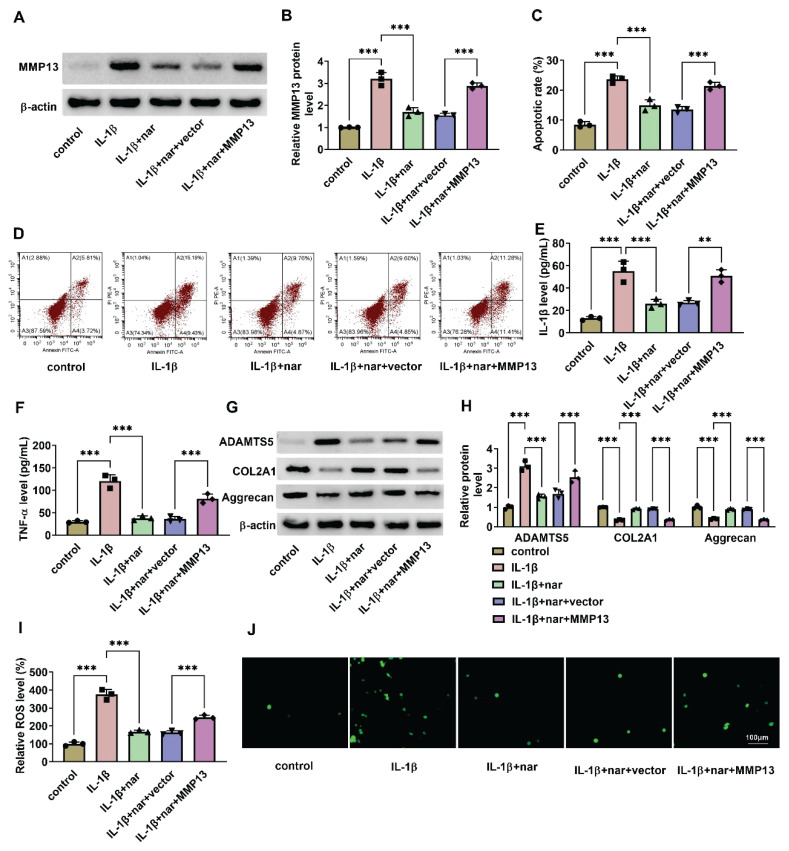
In IL-1β-induced C-28I2 cells, nar inhibits cell apoptosis, inflammation factor productions, ECM degradation, and ROS production *via* regulating MMP13. The cells were seeded in the microplates and then exposed to DMSO alone, IL-1β (10 ng/ml) alone, a combination of IL-1β (10 ng/ml) and nar (5 μM), or after being transfected with vector or MMP13, cells were treated with IL-1β (10 ng/ml) and nar (5 μM). MMP13 levels by Western blot (**A, B**), cell apoptosis by flow cytometry (**C, D**), TNF-α and IL-1β levels by ELISA assays (**E, F**), the levels of ADAMTS5, COL2A1, and Aggrecan by western blot (**G, H**), and ROS levels by commercial assay kit (**I, J**). ** *P*<0.01, *** *P*<0.001.

To further elucidate the underlying mechanism by which nar alleviates OA progression, the following experiments were conducted. As shown in Fig. 6A, Western blot assay was used to measure the expression of MMP13 in each group, the results showed that IL-1β increased MMP13 expression compared with the control group, but nar weakened this action. In addition, overexpression of MMP13 in combination with nar treatment promoted MMP13 levels when compared with the IL-1β+nar+vector group (Fig. 6B). Nar remitted IL-1β-induced promotion on cell apoptosis, which was abolished by MMP13 (Fig. 6C, D). When compared with the Control group, IL-1β and TNF-α productions were increased in the IL-1β group, but nar weakened this action; MMP13 overexpression could promote IL-1β and TNF-α levels when compared with the IL-1β+nar+vector group (Fig. 6E, F). IL-1β facilitated ADAMTS5 expression in C-28I2, and reduced COL2A1 and Aggrecan levels, which was abolished by nar; when compared with the IL-1β+nar+vector group, MMP13 promoted ADAMTS5 expression and inhibited COL2A1 and Aggrecan expression (Fig. 6G, H). Additionally, in IL-1β-stimulated cells, nar reduced ROS production; however, this action was abolished by MMP13 over-expression (Fig. 6I, J). Overall, nar curbs cell apoptosis, inflammation factor productions, ECM degradation, and ROS production in IL-1β-induced C-28I2 cells *via* reducing MMP13.

## Discussion

OA is the 15^th^ leading cause of disability globally, accounting for 2.2 % of total years of disability globally [15]. Currently, the pathogenesis of OA remains poorly understood and is regarded as a complex process that has not yet been fully elucidated. In clinical practice, symptom relief has become an effective strategy to treat OA. Thus, it is important to explore the potential mechanisms that affect the progress of OA.

The GSE283079 integrated dataset was analyzed to identify DEGs associated with OA, and then up-regulated DEGs were analyzed by GO enrichment analysis, which showed that these DEGs were associated with collagen metabolic processes, chondrogenesis, and leukocyte proliferation.

Nar demonstrates a broad array of pharmaco-logical activities, including anti-inflammatory, bone defect repair, anti-apoptotic, and antioxidant properties [16–18]. Currently, nar has been used to treat osteoporosis in traditional Chinese medicine [19]. In our study, network pharmacological analysis showed that nar may participate in the treatment of OA. Additionally, MMP13, MMP1, and PLA2G2A may be the key targets for the treatment of OA with nar. COL2A1 and the structural proteoglycan Aggrecan and increased MMP13 and ADAMTS5 were markers of the inflammation-induced aberrant metabolism of cartilage ECM, which leads to cartilage ECM degradation and exacerbates the progression of OA [20,21]. Moreover, it has been reported that MMP13 is a target gene in the progression of osteoarthritis [22]. In this study, the molecular docking results demonstrated that the binding energy of nar and MMP13 was strongest. Therefore, the authors suggested that nar remitted OA *via* targeting MMP13.

A previous study has shown that TNF-α and IL-1β play a central role in the pathological development of OA [23]. In the *in vitro* model of neuroinflammation model, nar suppressed TNF-α, IL-1β, and IL-6 expression [24]. Furthermore, in a mouse model of rheumatoid spondylitis, intraperitoneal administration of flavonoid drugs was able to reduce the expression of TNF-α, IL-1β, and IL-6 [25]. Notably, in our study, nar inhibited OA damage, apoptosis, TNF-α and IL-1β expression, and MMP13 and ADAMTS5.

MMPs, a class of matrix-degrading proteinases, are made by chondrocytes, neutrophils, and synovial fibroblasts and are involved in the formation of cartilage, osteogenesis, and the breakdown of extracellular matrix [26]. MMP13 is a member of MMP, a powerful enzyme that is highly specific for degrading type II collagen in the cartilage matrix, and the upregulation of MMP13 has been shown to be related to the development of OA [27,28]. The level of MMP13 immunoexpression in serum may be important in diagnosis, measurement of disease severity, and prediction of OA, but this assessment is appropriate for patients with advanced disease [29]. Wang *et al.* demonstrated that Wnt5a promoted cell apoptosis in chondrocytes *via* increasing MMP13 [30]. Additionally, levels of MMP13 mRNA and protein were increased in IL-1β-stimulated chondrocytes, and our findings were consistent with the findings of this study that MMP13 protein levels were promoted in IL-1β-stimulated chondrocytes [31]. IL-1β could upregulate the enzymes involved in cartilage matrix degradation, including MMP and ADAMTS5 [32,33]. Nar can improve the weight-bearing ability of rats with osteoarthritis and inhibit the key enzyme mediators (such as MMP-13 and ADAMTS-5) that are responsible for the progression of osteoarthritis in affected mice [34]. In IL-1β-induced chondrocytes, nar inhibited cell apoptosis, TNF-α and IL-1β levels, ECM degradation, and ROS production *via* decreasing MMP13. MMP13 may be regarded as a key mediator in nar treatment of OA.

Although our research results indicated that nar was expected to become effective in OA therapy. Currently, the effects of nar were mainly investigated through oral absorption. However, due to the large hydrophobic ring structure of Nar, its low solubility and low permeability result in limited bioavailability of Nar [35]. The bioavailability of oral administration of naringin is approximately 5–9 % [36]. Therefore, oral administration of Nar may still not be effective. Additionally, if administered by intravenous injection, nar will also undergo degradation during its circulation in the bloodstream. At present, *in vitro* attempts are made to enhance the bioavailability and absorption of flavonoids by increasing their solubility and dissolution rate, as well as to prevent intestinal degradation by encapsulating them in nanoparticles, microparticles or water-soluble fibers [37]. Mohanty *et al.* demonstrated that, compared with free nar, polymer nanoparticles loaded with nar can more effectively reduce the arthritis score of treated rats [38]. pH-Responsive Mesoporous Silica Nanoparticles Loaded with Nar, they promote bone regeneration and inhibit osteoclast activity *in vivo* [39]. Nar-bioglass hydrogels could retain the usual chondrocyte morphology, encourage macrophage polarization in the M2 types, efficiently inhibit ECM degradations, and restore the defects tissue cartilages [40]. Nar was integrated into an electrospun nanoscale scaffold composed of poly(ɛ-caprolactone) and poly(ethylene glycol)-block-poly(ɛ-caprolactone), and was used as a bone regeneration implant [41]. Zeolitic Imidazolate Framework-8 with Encapsulated Nar Synergistically has jointly enhanced the antibacterial and osteogenic properties of titanium implants, thereby promoting bone integration [42]. Overall, these studies suggest that nar may partially promote the repair of osteoarthritis through specific pathways. In future research, it will be explored whether the therapeutic effect of combining nar and MMP13 inhibitors treated OA progression by local intra-articular administration, incorporation into cartilage implants, or intra-articular injection formulations. It has the potential to become a valuable contribution to the field of degenerative joint disease and natural compound therapeutics.

Taken together, nar mitigates OA damage *in vivo*. Furthermore, IL-1β-induced C-28I2 cells, nar remits malignant activity through decreasing MMP13. These findings shed some light on the specific mechanisms by which nar exert a protective effect against OA and suggest that nar have therapeutic potential for the treatment of OA.

## Abbreviations

ACLT: anterior cruciate ligament transected
ADAMTS5: A disintegrin and metalloproteinase with thrombospondin 5
CCK8: Cell counting kit 8
COL2A1: Type II collagen alpha 1
DAPI: 4′,6-diamidino-2-phenylindole
DCFH-DA: 2′,7′-Dichlorodihydrofluorescein diacetate
DEGs: Differentially expressed genes
ECL: Electrochemi-luminescence
ECM: Extracellular matrix
GEO: Gene expression omnibus
GO: Gene Ontology
HE: Hematoxylin and Eosin
IL-1β: Interleukin-1β
MMP1: Matrix metalloproteinase 1
MMP13: Matrix metalloproteinase 13
Nar/nar: Naringin
OA: Osteoarthritis
OARSI: Osteoarthritis Research Society International
PLA2G2A: Phospholipase A2 group IIA
PPI: Protein-protein interaction
ROS: Reactive oxygen species
RSPO2: R-spondin 2
STRING: Search tool for the retrieval of interacting genes/proteins
TGF-β: Transforming growth factor-β
TNF-α: Tumor necrosis factor-α
TUNEL: Terminal deoxynucleotidyl transferase dUTP nick end labeling
